# Independent Shifts of Abundant and Rare Bacterial Populations across East Antarctica Glacial Foreland

**DOI:** 10.3389/fmicb.2017.01534

**Published:** 2017-08-10

**Authors:** Wenkai Yan, Hongmei Ma, Guitao Shi, Yuansheng Li, Bo Sun, Xiang Xiao, Yu Zhang

**Affiliations:** ^1^School of Life Sciences and Biotechnology, Shanghai Jiao Tong University Shanghai, China; ^2^SOA Key Laboratory for Polar Science, Polar Research Institute of China Shanghai, China; ^3^State Key Laboratory of Ocean Engineering, Shanghai Jiao Tong University Shanghai, China; ^4^Institute of Oceanography, Shanghai Jiao Tong University Shanghai, China

**Keywords:** succession, East Antarctica, glacial foreland, ice thickness, abundant and rare bacteria

## Abstract

Glacial forelands are extremely sensitive to temperature changes and are therefore appropriate places to explore the development of microbial communities in response to climate-driven deglaciation. In this study, we investigated the bacterial communities that developed at the initial stage of deglaciation using space-for-time substitution in the foreland of an ice sheet in Larsemann Hills. A series of soil samples across the glacial foreland were deeply sequenced with 16S rRNA gene amplicon sequencing to determine the bacterial community, including both abundant bacteria, which contribute more to geobiochemistry, and rare bacteria, which serve as a seed bank for diversity. Our results show that abundant bacterial communities were more sensitive to changing conditions in the early stages of deglaciation than rare community members. Moreover, among the environmental parameters tested, which included total organic carbon, pH, and moisture of the soils, ice thickness was the most influential factor affecting the community structure of abundant bacteria. These results show the different effects of abundant and rare bacteria on community shifts and highlight ice thickness as the primary factor affecting the bacterial community in the early stages of deglaciation. The response of microbial community to climate change can be predicted with more certainty in this polar region.

## Introduction

Worldwide deglaciation due to global warming has received much attention in the investigation of microbial diversity and adaptability in recent decades (Bradley et al., 2014). The deglaciation process exposes terrestrial ecosystems that have been previously locked under ice for thousands of years, providing a unique opportunity to explore the response of the microbial community to climate-driven environmental changes. With atmospheric exposure, the gradually exposed soil is subject to wind, solar radiation, rain, snow, and aeolian inputs of particles and organic materials; thus, the soil properties gradually change in the glacier foreland. The C, N and ion contents change because of melt water and increases in temperature (Zumsteg et al., 2012). Oxygen, light and radiation also increase after the covering ice is melted (Kaufmann, 2002), and with the dramatic daily temperature changes in the summertime, freeze-thaw cycles occur in the foreland (Kuhn, 2001). Understanding the changes in microbial communities in connection with soil exposure and environmental parameters can help to predict the response of bacterial communities to changing environmental conditions during the deglaciation process.

Previous microbiological investigations revealed that active microbial communities occur in glacial forelands and are likely supported by processes of biogeochemical transformation (Wadham et al., 2004). With the application of culture dependent and independent methods, many studies have investigated microbial activity and diversity in glacial forelands (Sigler et al., 2002; Nemergut et al., 2007). Based on long soil chronosequences, some abundant taxa are correlated with soil moisture, pH and ion concentration, as shown for the terminus of the Fox and Franz Josef glacier (Foght et al., 2004), the two forelands of the East Antarctic glacier (Bajerski and Wagner, 2013), the Damma glacial foreland in central Switzerland (Zumsteg et al., 2012) and other glacial forelands (Nicol et al., 2005; Nemergut et al., 2007; Lazzaro et al., 2009; Schütte et al., 2009; Bárcena et al., 2010). However, there is no report about the community change in the initial stage of deglaciation, using a shorter soil chronosequence. Additionally, most analyses of community structure are focused on abundant taxa and do not include rare taxa because of the limitation from older sequencing methods, such as Sanger (Ansorge, 2009). Rare bacteria can bloom and play important roles in community function (Sauret et al., 2014) and are studied in various environments, including coastal sands (Gobet et al., 2012), the Arctic Ocean (Galand et al., 2009) and the deep sea (Sogin et al., 2006). Therefore, more studies are required to investigate the rare taxa of the glacial foreland, particularly in the initial stage of deglaciation. This component of the microbial community is important for further community development (Wittebolle et al., 2009) and response to climate change (Bradley et al., 2014).

East Antarctica proglacial regions have extremely low bioavailability of water and nutrients, cold temperatures and frequent freeze-thaw cycles (Verleyen et al., 2011), therefore present harsher living conditions compared to mountain glaciers (Thompson, 2009) and proglacial regions in Arctic (Breen and Levesque, 2007), west Antarctica (Bromwich et al., 2013) and the Antarctic Peninsula (Davies et al., 2014). Studies that investigate the effects of deglaciation on bacterial community structure remain comparatively recent and are scarce in East Antarctica regions (Bajerski and Wagner, 2013). In this study, a strategy of space-for-time substitution was employed to study the bacterial 16S rRNA gene diversity in the glacier foreland in Larsemann Hills, East Antarctica. This is the first study about bacterial diversity utilizing next generation sequencing in this area. Soils from the short-distance gradient were sampled to substitute for the early stages of deglaciation in the glacier foreland. The bacterial community, including abundant and rare bacterial groups, was characterized using Illumina MiSeq sequencing, and components of the community were correlated with geochemical factors to study the response to environmental change. The following two questions were addressed in this study: (1) Did abundant and rare bacteria respond differently to the change in conditions with deglaciation? (2) What primary factor affected the bacterial community structure in the early stages of deglaciation? With answers to these questions, we might gain a better understanding of the changes that occur in bacterial community structure, including those in both abundant and rare groups, during the transition period from the subglacial to the proglacial condition.

## Materials and Methods

### Study Site and Sample Description

Soil samples were collected from the glacial foreland in Larsemann Hills in East Antarctica (-69.39762S, 76.40666 E). The samples were collected during the 29th Chinese National Antarctic Research Expedition in the Antarctic summer in February 2013. In total, five samples were collected across 2 m near the ice sheet. Site 1 and site 2 were ice-free when sampled, and their surface layers of soil, approximately 5 cm, were collected. Sites 3, 4 and 5 were covered by ice. The covering ice was gently cracked and the ice fractures were removed before sampling the soil beneath. These five samples were all frozen during sampling. The samples were stored in plastic bags and kept at -20°C during transport and storage in the laboratory until they were used for further analysis. Each soil sample was homogenized and sub-sampled for DNA extraction and geochemical measurements.

### Geochemical Data Analyses

Soil pH was measured in a soil extract (Bajerski and Wagner, 2013). The anions in the soil extract were measured with chromatography (MIC, Metrohm, Herisau, Switzerland) (Ankley and Schubauer-Berigan, 1994). The moisture content of approximately 20 g of soil was determined by weighing the soil before and after freeze-drying (Bajerski and Wagner, 2013). The total carbon content was determined using an automatic element analyzer (TOC-VCPN system; Shimadzu, Japan).

### DNA Extraction and Bacterial 16S rRNA Gene Amplification

An SDS-based method was employed to extract the DNA from soil (Natarajan et al., 2016). The bacterial V4 region of the 16S rRNA gene was amplified with a special bacterial primer pair 533F (TGCCAGCAGCCGCGGTAA)/Bact806R (GGACTACCAGGGTATCTAATCCTGTT) (Hongoh et al., 2003; Klindworth et al., 2013). A sample tagging approach was employed, and a different barcode was added before the forward primer for each sample. The PCR reagents were mixed as follow: 5 μl of 10× Taq buffer (Takara, Otsu, Shiga, Japan), 4 μl of dNTP (Takara, Otsu, Shiga, Japan), 1 μl of each primer (10 μM stored concentration), 0.25 μl of Ex Taq DNA polymerase (Takara, Otsu, Shiga, Japan), approximately 50 ng of DNA, 2.5 μl of BSA (Bull Serum Albumin), and 32.75 μl of water. The PCR amplification consisted of an initial denaturation at 94°C for 5 min; 25 cycles of denaturation at 94°C for 40 s, annealing at 58°C for 40 s, and extension at 72°C for 1 min; and a final extension at 72°C for 8 min. The PCR products were purified with a Gel Extraction Kit (Omega Bio-Tek, Norcross, GA, United States) according to the manufacturer’s instructions. The reads were obtained with MiSeq sequencing platform (Illumina, San Diego, CA, United States).

### Quantification of Bacterial 16S rRNA Gene Copy Number

qPCR was employed to quantify the bacterial 16S rRNA gene copy numbers based on the method reported before (Nadkarni et al., 2002). For a standard sample, the bacterial 16S rRNA gene (HQ340606.1) was amplified with primers 331F/797R (Nadkarni et al., 2002) and cloned into vector T. Then, the 16S rRNA gene fragment was sequenced and analyzed. The plasmid with the 16S rRNA gene clone was 3163 bp, was transformed into competent DH 5α cells (Trans gene, Beijing, China) and was then extracted in abundance. The concentration of the plasmid was quantified with a spectrophotometer (Nanodrop 2000, ThermoFisher). The copy number of the 16S rRNA gene was calculated according to the concentration of the plasmid as the copy number μl^-1^ DNA = 6.02 × 10^23^× C(g μl^-1^) × 660^-1^ × 3163^-1^ bp. The plasmid DNA was diluted 10-fold in ultrapure water (MILLIPORE) to create a dilution series from 8.86 × 10^8^ to 8.86 × 10^2^ copies per μl.

The SYBR green fluorophore was employed to perform qPCR with 10 μl of SYBR green Prim Mix Taq II (2x) (Takara, Otsu, Shiga, Japan), 0.4 μl of RoxReference Dye II (50x) (Takara, Otsu, Shiga, Japan), 1 μl of each primer 331F/797R (10 μM), 6.6 μl of ultrapure water (MILLIPORE) and 1 μl of DNA. The samples and standard sample were amplified in the same plate in triplicate. The qPCR was run on a Fast Real-Time PCR System (ABI 7500) with the following PCR conditions: 30 s at 95°C, followed by 40 cycles of denaturation at 95°C for 5 s, annealing at 55°C for 30 s, and extension at 72°C for 1 min. Image capture was conducted upon annealing. A melt curve analysis was performed to ensure the specific amplification with the following conditions: 95°C for 15 s, 60°C for 1 min and temperature increases of 0.5°C increments every 10 s from 60°C to 95°C. Images were captured during the period of temperature increase. The amplification efficient of standard curve: *R*^2^= 0.998, eff % = 105.

### Data Processing and Statistical Analyses

The raw sequence data were filtered using quality control steps reported before (Zhang et al., 2016b). The chimera sequences and non-bacterial sequences were removed with QIIME and the Ribosomal Database Project (RDP) (Caporaso et al., 2010). To eliminate the effect of different sequencing depths, sequences were rarefied to even depth by random sampling using QIIME and 18 000 reads were obtained for each sample. To reduce artificial error, singletons were removed (Jones and Lennon, 2010) from the five samples. Rare and abundant OTUs were arbitrarily defined as: abundant: >1%, and rare: <0.1% (Pedrós-Alió, 2006). Taxa between 0.1 and 1% abundance were defined as a middle group. Since the cutoffs used to define rare taxa may affect the main results presented in this study, we tested the cutoff 1% besides 0.1% to clarify that the significance of the results are unlikely artifacts. The coefficient of variation is calculated as the ratio of the standard deviation to the mean abundance of one family in five sites. The R statistical environment (R version 3.1.2, R Development Core Team, 2009) was employed to perform non-metric multidimensional scaling (NMDS) using Bray-Curtis distance and perform redundancy analysis (RDA) using Euclidean distance with the Vegan package (Oksanen et al., 2009). Pearson correlation coefficients for taxa abundance and environmental factors were calculated. The heat map was created with the gplots package (Bolker et al., 2009) within R. The co-occurrence analysis was performed with R using the Spearman correlation coefficient. Abundant OTU sequences were submitted to the NCBI database with accession numbers KX094445-KX094469. All sequence data were deposited in the National Center for Biotechnology Information (NCBI) Sequence Read Archive with the accession number SRP095246.

## Results

### Study Site Characteristics

**FIGURE 1:**
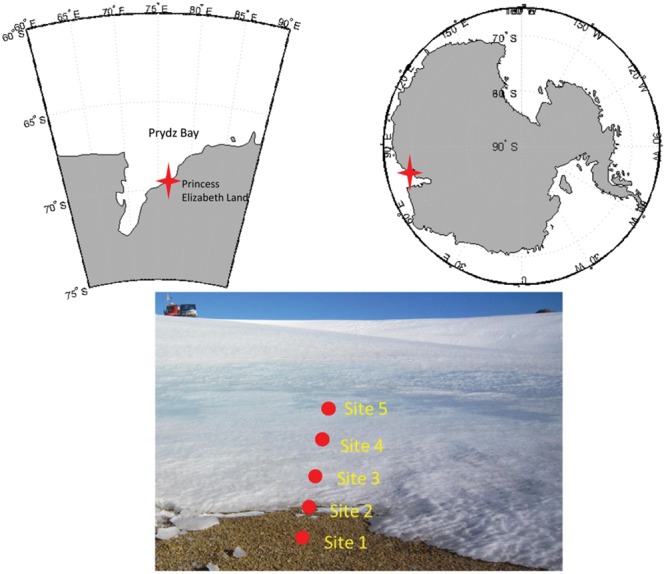
Maps of the study site and the locations of the sampling sites in the glacial foreland. The study site was near the Russian Progress Station at Larsemann Hills on the shore of Prydz Bay. Five sites, including three ice-covered sites, were sampled.

Samples were collected from the glacier foreland in Larsemann Hills, East Antarctica (**Figure 1**). Samples were taken beneath the ice sheet, which was approximately 5–40 cm thick (**Table 1**) in the foreland. The soil was completely frozen when sampled. This transect represented the initial stage of deglaciation.

**Table 1:** Biogeochemical data for soils in the glacial foreland.

			Soil				Soil Extraction				
Sample ID	Distance to ice sheet margin (cm)	Covered ice thickness (cm)	Total carbon (%)	Inorganic carbon (%)	Total organic carbon (%)	Moisture (%)	pH	F^-^ nmol g^-1^ soil	Cl^-^ nmol g^-1^ soil	NO_3_^-^ nmol g^-1^ soil	SO_4_^2-^ nmol g^-1^ soil
S1	50	0	0.053	0.002	0.051	5.16	6.09	25.7	42.7	20.6	9.0
S2	0	0	0.051	0.002	0.049	4.58	6.26	27.7	24.8	25.1	8.9
S3	–50	20	0.041	0.003	0.038	10.56	6.43	13.6	121.0	17.3	15.4
S4	–100	30	0.066	0.002	0.064	7.75	6.05	43.9	50.1	26.1	17.2
S5	–150	40	0.036	0.002	0.033	5.92	5.68	18.8	59.5	18.6	8.9

As shown in **Table 1**, the soil moisture at site 2 was the lowest with only 4.58% while that at site 3 was the highest with 10.56%. The TOC contents at different sites also varied, with the lowest value of 0.033% at site 5 and the highest value of 0.064% at site 4 (**Table 1**). The IC contents were generally very low and the soil pH was below 7, which is likely due to the silicate soil character at this location. The NO_3_^-^ and SO_4_^2-^ concentrations in the soil extractions ranged from approximately 8.9–26.1 nmol g^-1^ of soil (**Table 1**). No PO_4_^3-^ was detected. Site 3 contained the highest concentration of Cl^-^ and lowest concentration of F^-^ and NO_3_^-^ compared with those at other sites (**Table 1**). From each sampling site, 1–2 μg DNA per gram of soil was extracted. The bacterial 16S rRNA gene copy numbers were in a range of 3.48 × 10^8^ to 7.88 × 10^8^ copy g^-1^ of soil (**Table 2**). There was no significant Pearson correlation (*p*-value > 0.05) between distance to ice sheet margin and other factors including total organic carbon, moisture, pH and ions, indicating heterogeneity in the glacial foreland soils.

**Table 2:** Summary of the biomass indicators and the diversity indexes.

Sample ID	DNA ng g^-1^ soil	Bac16s rRNA Gene Copy g^-1^ soil	Reads-after evening sequencing depth	Reads-after removing singletons	Shannon	Chao1	Coverage	Total OTU	Abundant OTU	Rare OTU	Gini
S1	1220.61	4.03 × 10^8^	18000	17574	7.27	2040.76	0.9675657	1383	16	1262	0.89072
S2	1254.37	6.48 × 10^8^	18000	17587	7.34	2038.90	0.9675897	1359	18	1236	0.89336
S3	2168.92	7.88 × 10^8^	18000	17625	7.24	1972.08	0.9693617	1318	17	1190	0.89737
S4	1785.87	2.63 × 10^8^	18000	17570	7.21	2082.27	0.9680137	1333	17	1201	0.89635
S5	1009.24	3.48 × 10^8^	18000	17527	7.14	1994.62	0.9678781	1363	14	1236	0.89482

### Bacterial Community Structure in the Glacial Foreland

In total, 144 449 high-quality sequences were obtained after quality control. Sequence coverage reached 96% (**Table 2**). Singletons (which appeared once in the sequence data of the five samples) were eliminated. The OTU (97% similarity) fraction was grouped into abundant OTUs (abundance > 1%), middle group and rare OTUs (abundance < 0.1%) (Sogin et al., 2006; Gobet et al., 2012). In all five samples, the rare group accounted for 20.5–21.3% of the sequences (Supplementary Table S1) and 90.1–91.3% of the OTUs. The middle group accounted for 29.1–32.5% of the sequences (Supplementary Table S1). With another tested cutoff (abundant: > 1%, and rare: < 1%), the rare group accounted for 49.8–53.1% of the sequences (Supplementary Table S2). The soil DNA content was highest at site 3 at 2168.92 ng g^-1^, which was consistent with the highest bacterial 16S rRNA gene copy number of the five study sites. At site 3, the Chao1 index was the lowest (1972.08), and the Gini index was the highest (0.89737) among the five sites. The Shannon index of the five sites ranged from 7.14 to 7.34 (**Table 2**).

**FIGURE 2:**
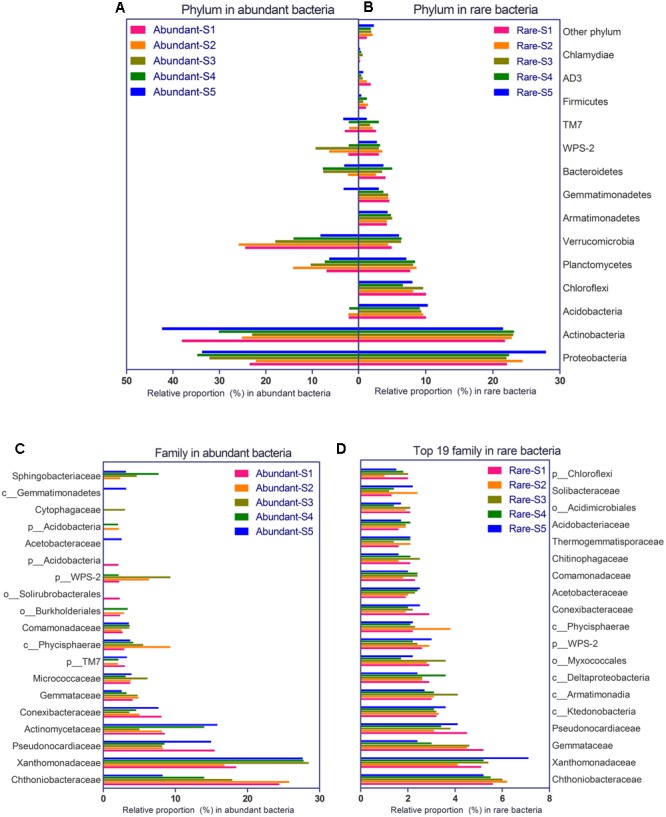
Phylogenetic composition of abundant bacteria (>1% frequency) and rare bacteria (<0.1% frequency) at the five study sites of the glacial foreland. **(A)** Phylum composition for abundant bacteria. **(B)** Phylum composition for rare bacteria. **(C)** Family composition for abundant bacteria. **(D)** Family composition for rare bacteria (193 total families were found in the rare bacteria, and the top 19 families (ranked by the average proportion in the five sites) are shown here).

The bacterial community consisted of 29 phyla and 193 families in the glacial foreland. For abundant bacteria, *Proteobacteria, Actinobacteria, Planctomycetes, Verrucomicrobia*, and *Bacteroidetes* were the dominant phyla (**Figure 2A**). More phyla of rare bacteria were found, including *Acidobacteria, Chloroflexi, Armatimonadetes*, and *Gemmatimonadetes* (**Figure 2B**). At the family level, *Chthoniobacteraceae, Xanthomonadaceae, Pseudonocardia*, and *Gemmataceae* were dominant in both abundant and rare bacteria groups (**Figures 2C,D**). More families were found for rare bacteria.

The community of abundant bacteria in soils of the ice-free sites was different from that in the soils of ice-covered sites (**Figure 2A**). At the phylum level, more *Verrucomicrobia* were found in the ice-free sites (unpaired *t*-test, *p*-value = 0.0248), whereas more *Proteobacteria* were found in the ice-covered sites (unpaired *t*-test, *p*-value = 0.0011) (**Figure 2A**). At the family level, more *Chthoniobacteraceae* were found in the ice-free sites (unpaired *t*-test, *p*-value = 0.0243), whereas more *Xanthomonadaceae* were found in the ice-covered sites (unpaired *t*-test, *p*-value = 0.0003) (**Figure 2C**).

### The Abundant Bacterial Community Structure Was More Diverse at the 5 Study Sites

**FIGURE 3:**
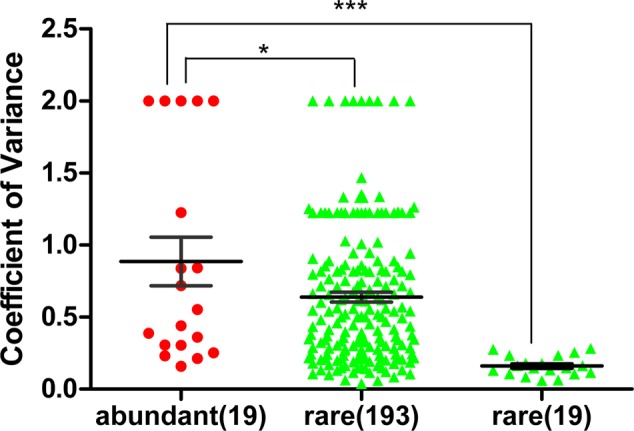
Coefficient of variation of abundant families and rare families at the five study sites (abundant OTU: >1%, and rare OTU: <0.1%). All 19 families of abundant bacteria, all 193 families of rare bacteria and the top 19 families of rare bacteria (ranked by the average proportion in the five sites) at the five study sites were analyzed. The mean coefficient of variation of the abundant families was significantly higher than that of the rare families (unpaired *t*-test, ^∗∗∗^*p* < 0.0001; ^∗^*p* < 0.05). Bar: mean ± SEM.

**FIGURE 4:**
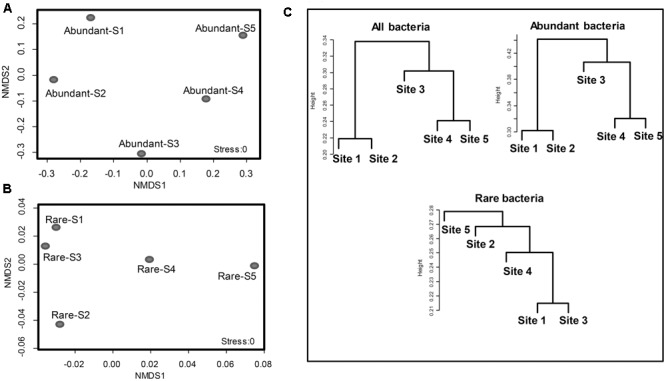
Clusters of abundant bacteria (>1%) and rare bacteria (<0.1%) as an indication of community structure. Non-metric multidimensional scaling (NMDS) of Bray-Curtis similarities of abundant **(A)** and rare **(B)** bacterial communities at the family level in the glacial foreland. **(C)** A dendrogram analysis of the communities of all bacteria, abundant bacteria and rare bacteria at the family level in the five glacial foreland soils.

The coefficient of variation for the families of abundant OTUs (19 families in total) among the five sites was compared with that of rare OTUs. The coefficient of variation for families of abundant OTUs was 0.8864, which was significantly higher than that of the top 19 families of rare OTUs (0.1614) (unpaired *t*-test, *p*-value < 0.0001; **Figure 3**) and the all families of rare OTUs (0.6390) (unpaired *t*-test, *p*-value < 0.05, **Figure 3**). Similar results were obtained with another tested cutoff (abundant: > 1%, and rare: < 1%, Supplementary Figure S1). The NMDS results showed different patterns between abundant bacteria and rare bacteria (**Figures 4A,B**). Site1 and site 3 were closer in rare bacteria while site 1 and site 2 were closer in abundant bacteria (**Figures 4A,B**). Site 1 and site 2 were also closer in middle group (Supplementary Figure S2). Combining the results for coefficient of variation and NMDS, the differences among the five gradient samples were larger for abundant bacteria than for rare bacteria, indicating that the rare group was less sensitive to deglaciation. The dendrogram analysis showed that the cluster pattern of abundant bacteria was similar to the pattern for all bacteria (**Figure 4C**), which indicated that the abundant bacteria contributed more to community structure differences than the rare bacteria due to their higher populations. Different cluster patterns between abundant bacteria and rare bacteria were also observed with another tested cutoff (<1%, Supplementary Figure S3).

### Abundant Bacteria Response to Geochemical Factors

**FIGURE 5:**
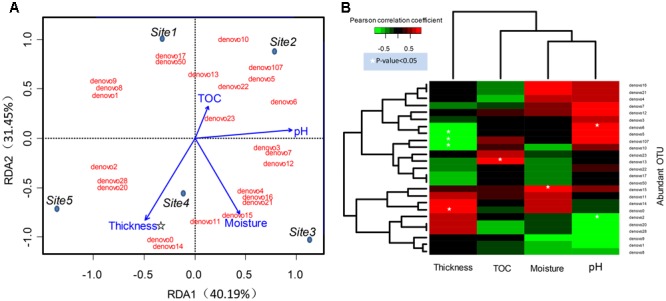
Relations between abundant OTUs and environmental factors. **(A)** Ordination diagram of the RDA of abundant bacteria OTU profile in the glacial foreland. Blue dots indicate the study sites. Sites 1 and 2 were ice-free, and Sites 3, 4, and 5 were ice-covered. Red text indicates the abundant bacteria OTUs. Blue arrows indicate the environmental variables. The star indicates that the *p*-value < 0.05 (Monte Carlo permutation test, Supplementary Table S3). **(B)** The environmental factors associated with abundant bacteria OTUs in the glacial foreland soil. The Pearson correlation coefficient indicates the correlation between OTU sequence abundance and an environmental factor. A white star indicates that the *p*-value of the correlation is <0.05.

**FIGURE 6:**
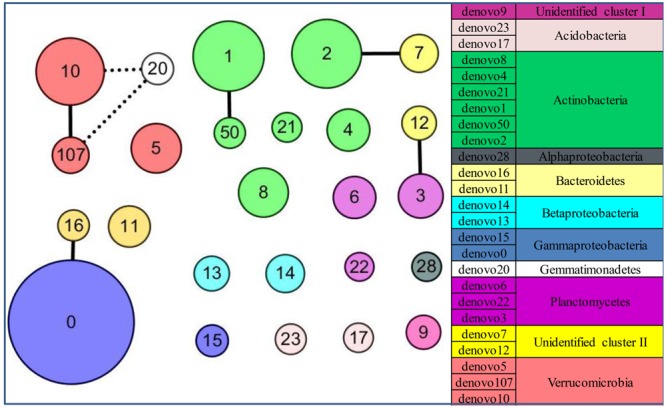
The patterns of keystone taxa for the community structure in the glacial foreland. Network of co-occurrence of abundant OTUs based on Spearman correlation analysis. The size of each node is proportional to the average abundance at the five sites. A connection stands for a strong (coefficient > 0.75 marked with a solid line or coefficient < –0.75 marked with a dashed line) and significant (*p*-value < 0.05) correlation.

The community structure of abundant bacteria was affected by environmental factors (**Figure 5**). The correlations between the abundance of abundant OTUs and geochemical factors were analyzed, and the results are shown in **Figure 5B**. Acting as the keystone taxa in community structure, OTUs denovo1 (*Actinobacteria*), denovo2 (*Actinobacteria*), denovo10 (*Verrucomicrobia*) and denovo0 (*Gammaproteobacteria*) had the most connections with other OTUs (Spearman’s correlation) and were at the same time highly abundant (**Figure 6**). The abundance of keystone taxa changed across the glacial foreland (Supplementary Figure S4) because of the influence of environmental factors. Ice thickness was the primary environmental factor that affected abundant bacterial community structure (**Figure 5A**, *r*^2^ = 0.9799, *p*-value < 0.05, Monte Carlo permutation test, Supplementary Table S3) and rare bacterial community structure (Supplementary Table S4). The distance to ice sheet margin showed no significant effect on abundant bacterial community structure (Supplementary Figure S5 and Table S3). The taxonomy of abundant OTUs was analyzed by constructing a phylogenetic tree (Supplementary Figure S6).

## Discussion

### The Ecosystem in the Glacial Foreland Is Sensitive to Climate Changes

The habitats formed by deglaciation in Larsemann Hills are an example of primary succession, with the result that soil characteristics were heterogeneous across the glacial foreland (**Table 1**). These study sites were also affected by the extreme environmental conditions of continental Antarctica (Bajerski and Wagner, 2013), and specifically, at our study sites, conditions were influenced by the glacier and snowcaps. Across the glacial foreland, no trends in soil water content, organic matter content, pH or ion concentration were observed because these parameters are influenced by strong winds, the snow melting process or the mechanical movement and downwash of clay (Schütte et al., 2009; Bajerski and Wagner, 2013). The local-scale variability and local microclimates can also influence habitat formation in Antarctica (Cannone et al., 2008). At site 3, the moisture, concentration of several ions, DNA content and the number of bacterial 16S rRNA gene copies were higher than those at any other sites (**Tables 1, 2**), which indicated heterogeneity in the glacial foreland. Only the ice thickness showed a gradient change across the glacial foreland at the five study sites (**Table 1**). Glacial movement can influence the ice thickness above the soil in the glacial foreland (Crossman et al., 2013). Furthermore, ice thickness in the glacial foreland is also affected by local snowfall and melt and further deglaciation (Bradley et al., 2014). At our study sites, the sampling sites were at the margin of the ice sheet, and the distance between the sites was 50 cm. This gradient reflected the change in conditions at the early stages of deglaciation and is therefore a promising model system to study the response of the microbial community to the early stages of deglaciation using the strategy of space-for-time substitution.

### Rare and Abundant Bacterial Community Structure in the Glacial Foreland

Did abundant and rare bacteria respond differently to the change in conditions with deglaciation?

The community characteristics of the rare and abundant bacteria were affected differently by changing conditions in the glacial foreland. The communities of total bacteria were clustered in ice-free sites (sites 1 and 2) separate from those in ice-covered sites (sites 3, 4, and 5; **Figure 4B**). The communities of abundant bacteria showed the same cluster pattern. However, the cluster pattern for the rare bacterial communities showed no correlation with ice coverage at the sites. The same cluster pattern between total bacteria and abundant bacteria indicated that the abundant bacteria contributed more to community structure differences than did the rare bacteria in the glacial foreland. Similar cluster results between total bacteria and abundant bacteria are also found in the Arctic Ocean (Galand et al., 2009) and at deep-sea hydrothermal vents (Anderson et al., 2014).

Compared with the community structure of total bacteria among the five study sites, the family abundance of the abundant bacteria showed more heterogeneity than that of the rare bacteria (**Figure 2**). To reduce the effect of the difference in abundance between abundant bacteria and rare bacteria, the coefficient of variation was used to analyze the heterogeneity of the families in the two groups. The heterogeneity in abundant bacteria was significantly higher than that in rare bacteria (*p*-value < 0.05, **Figure 3**). Based on these results, at the early stages of deglaciation, the community of abundant bacteria responded more strongly to the changed conditions than the community of rare bacteria.

### What Primary Factor Affected the Bacterial Community Structure in the Early Stages of Deglaciation?

Ice thickness had the most significant effect on community structure (**Figure 5**).

The heterogeneity of the geochemical parameters influenced the structure of abundant bacterial community members (**Figure 5**). The correlations of some abundant phylotypes with soil moisture, pH and TOC content were found here (**Figure 5B**). The keystone taxon OTU denovo2 was correlated with pH (**Figure 5B**). The keystone taxa OTUs denovo2 and denovo1 were members of *Actinobacteria*, which are typical soil bacteria, including in glacial foreland soils (Zhang et al., 2016a). *Actinobacteria* have important roles in soil development and the cycling of carbon, nitrogen and other elements (Goodfellow and Williams, 2003). *Actinobacteria* may also influence other microbes by producing extracellular hydrolytic enzymes (Eisenlord and Zak, 2010) and antibiotics (Berdy, 2012). *Actinomycetales* was also found as the dominant taxa and was affected by soil pH in a previous study (Zhang et al., 2016a). Additionally, soil pH, moisture and soil formation is affected by the deglaciation process and snowfall or melt (Hodson et al., 2008; Finn et al., 2010; Bajerski and Wagner, 2013; Bradley et al., 2014). Additionally, ecosystem development also effects the TOC content (Bernasconi et al., 2011). Based on longer distances (i.e., longer glacial recession times), soil moisture, pH and conductivity showed effect on *Cyanobacteria, Bacteroidetes* and *Deltaproteobacteria* in a previous study of the glacier foreland in East Antarctica (Bajerski and Wagner, 2013).

However, in the early stages of deglaciation, the structure of abundant bacterial community was most affected by the ice thickness (**Figure 5A** and Supplementary Table S3). The ice thickness and distance to ice sheet margin were in gradients along this transect (**Table 1**). Distance-decay relationships with bacterial communities were reported in some long soil chronosequences (Schütte et al., 2009; Zumsteg et al., 2012; Franzetti et al., 2013). However, in this short soil chronosequence, distance showed no significant effect on abundant bacterial community structure (Supplementary Figure S5 and Table S3). The bacterial communities in ice-covered sites were different from that in ice-free sites in the foreland of the glacier (**Figures 2, 4B**). The keystone taxon denovo10 was negatively correlated with ice thickness (**Figure 5B**), and more of this OTU was found in ice-free sites. OTU denovo10 was related to the strain *Chthoniobacter flavus* Ellin428, which is an aerobic heterotrophic bacterium that requires oxygen for growth (Sangwan et al., 2004). In those sites covered with ice, oxygen was likely limited because of isolation by frozen ice above the soil, with possible effects on aerobic heterotrophic bacteria. The keystone taxon denovo10 belonged to *Verrucomicrobia*, which are ubiquitous in soil (Zhang and Xu, 2008). Most *Verrucomicrobia* are mesophilic (Sangwan et al., 2004) and saccharolytic (Janssen, 1998). The class *Spartobacteria* is dominant in soils (Bergmann et al., 2011), and although most of these bacteria remain uncultivated, some show adaptation to low substrate concentrations (Wagner and Horn, 2006). These bacteria could have an important role in community development in the barren glacial foreland due to their oligotrophic life history strategy (Senechkin et al., 2010; Bergmann et al., 2011). Based on the V4 region of the 16S rRNA gene, the keystone taxon denovo0 was highly identical to *Rhodanobacter ginsengisoli* GR17-7 (Supplementary Figure S6), which is in the *Gammaproteobacteria*. More denovo0 was found in the ice-covered sites (25–27%) than in the ice-free sites (16–18%). *Rhodanobacter ginsengisoli* GR17-7 was isolated from a ginseng field, and these bacteria are Gram-negative, motile, aerobic rods with an optimal temperature of 28°C (Weon et al., 2007). More studies regarding this strain are required to explain the correlation with ice thickness. Ice thickness in the glacial foreland influences the oxygen availability and temperature perturbation in soil (Hodson et al., 2008), in addition to the pressure from some larger glaciers (Hooke et al., 1990), which likely has a major effect on the structure of microbial communities in the early stages of deglaciation.

### Bacterial Community Composition and Stability in the Glacial Foreland

Abundant and rare bacteria contributed to community composition and stability as conditions changed during succession in the glacial foreland. Abundant bacteria are regarded as the primary group to mediate geochemical cycles (Pedrósalió, 2012). As habitat conditions changed during the deglaciation, the abundant bacteria were affected (**Figure 4A**); for example, *Chthoniobacter flavus* Ellin428-related bacteria were affected by ice thickness and their role in the transformation of organic carbon compounds in soil (Sangwan et al., 2004) may mediate the carbon cycle in the glacial foreland. Rare bacteria showed less sensitivity to changes in habitat conditions and therefore exhibited the characteristic of a seed bank to maintain community diversity and stability. Therefore, rare bacteria can contribute to community diversity and enhance ecosystem reliability (Naeem and Li, 1997) and function (Cardinale et al., 2002; Bell et al., 2005). Additionally, some conditionally rare bacteria can increase rapidly in abundance and further affect geochemical cycles, such as during massive deep-sea oil discharges in the Gulf of Mexico (Kleindienst et al., 2016) or during a soil rewetting event (Aanderud et al., 2015). Blooms of rare bacteria may also occur on the longer soil chronosequences of glacial forelands in adaptation to larger changes in conditions, which contribute to maintaining community stability.

## Conclusion

In conclusion, by employing the strategy of space-for-time substitution, the succession of the bacterial community was studied in the glacier foreland in Larsemann Hills, East Antarctica. The abundant bacteria were more sensitive to the changes in conditions and were most affected by the ice thickness. The rare bacteria preserved more than 90% of the total bacterial OTUs in the harsh environment, which contributed to the maintenance of community stability. This study helps to understand the response of microbial communities to changing conditions due to deglaciation in Polar Regions by highlighting the different effects of abundant and rare bacteria on community shifts.

## Author Contributions

WY, HM, and YZ designed the experiments; WY performed the lab experiments; GS sampled the soil from East Antarctica. YL and BS provided the field and logistical support and theoretical guidance. WY, YZ, XX, and HM analyzed the data and wrote the manuscripts.

## Conflict of Interest Statement

The authors declare that the research was conducted in the absence of any commercial or financial relationships that could be construed as a potential conflict of interest.
