# Supplementary material for: Independent Shifts of Abundant and Rare Bacterial Populations across East Antarctica Glacial Foreland
Source: Front Microbiol. 2017 Aug 10;8:1534. doi: 10.3389/fmicb.2017.01534 (PMC5554324; doi:10.3389/fmicb.2017.01534)
Supplement: Supplementary file 1 [file Table_1.DOCX]

Supplementary Information

Independent shift of abundant and rare bacterial populations across the glacial foreland in East Antarctica

Wenkai Yan^1^, Hongmei Ma^2*^, Guitao Shi^2^, Yuansheng Li^2^, Bo Sun^2^, Xiang Xiao^1^, Yu Zhang^3*^

^1^ School of Life Sciences and Biotechnology, Shanghai Jiao Tong University, Shanghai, China

^2^ SOA Key Laboratory for Polar Science, Polar Research Institute of China, Shanghai, China

^3^ State Key Laboratory of Ocean Engineering, Shanghai Jiao Tong University, Shanghai, China

*** Correspondence:**

*Yu Zhang: zhang.yusjtu@sjtu.edu.cn*

*or Hongmei Ma: mahongmei@pric.org.cn*

Table S1. The sequence percentages of abundant (frequency>1%), middle (1%> frequency >0.1%), and rare (frequency<0.1%) groups.

| Group | S1 | S2 | S3 | S4 | S5 |
| --- | --- | --- | --- | --- | --- |
| Abundant | 48.8% | 49.5% | 50.2% | 49.7% | 46.9% |
| Middle | 29.1% | 29.1% | 29.1% | 29.8% | 32.5% |
| Rare | 22.1% | 21.3% | 20.7% | 20.5% | 20.6% |
