# Supplementary material for: Independent Shifts of Abundant and Rare Bacterial Populations across East Antarctica Glacial Foreland
Source: Front Microbiol. 2017 Aug 10;8:1534. doi: 10.3389/fmicb.2017.01534 (PMC5554324; doi:10.3389/fmicb.2017.01534)
Supplement: Supplementary file 3 [file Table_3.DOCX]

Supplementary Information

Independent shift of abundant and rare bacterial populations across the glacial foreland in East Antarctica

Wenkai Yan^1^, Hongmei Ma^2*^, Guitao Shi^2^, Yuansheng Li^2^, Bo Sun^2^, Xiang Xiao^1^, Yu Zhang^3*^

^1^ School of Life Sciences and Biotechnology, Shanghai Jiao Tong University, Shanghai, China

^2^ SOA Key Laboratory for Polar Science, Polar Research Institute of China, Shanghai, China

^3^ State Key Laboratory of Ocean Engineering, Shanghai Jiao Tong University, Shanghai, China

*** Correspondence:**

*Yu Zhang: zhang.yusjtu@sjtu.edu.cn*

*or Hongmei Ma: mahongmei@pric.org.cn*

Table S3. Monte Carlo permutation test for the effect of factors on abundant community structure (Number of permutations: 120).

| Factor | RDA1 | RDA2 | r^2^ | p-value |
| --- | --- | --- | --- | --- |
| TOC | 0.38023 | 0.92489 | 0.1278 | 0.87500 |
| Thickness | -0.52129 | -0.85338 | 0.9799 | 0.01667 |
| pH | 0.9963 | 0.08592 | 0.9704 | 0.06667 |
| Moisture | 0.50092 | -0.86549 | 0.8286 | 0.15833 |
| Distance | 0.49508 | 0.86885 | 0.8884 | 0.16667 |
