# Supplementary material for: Independent Shifts of Abundant and Rare Bacterial Populations across East Antarctica Glacial Foreland
Source: Front Microbiol. 2017 Aug 10;8:1534. doi: 10.3389/fmicb.2017.01534 (PMC5554324; doi:10.3389/fmicb.2017.01534)
Supplement: Supplementary file 4 [file Table_4.DOCX]

Supplementary Information

Independent shift of abundant and rare bacterial populations across the glacial foreland in East Antarctica

Wenkai Yan^1^, Hongmei Ma^2*^, Guitao Shi^2^, Yuansheng Li^2^, Bo Sun^2^, Xiang Xiao^1^, Yu Zhang^3*^

^1^ School of Life Sciences and Biotechnology, Shanghai Jiao Tong University, Shanghai, China

^2^ SOA Key Laboratory for Polar Science, Polar Research Institute of China, Shanghai, China

^3^ State Key Laboratory of Ocean Engineering, Shanghai Jiao Tong University, Shanghai, China

*** Correspondence:**

*Yu Zhang: zhang.yusjtu@sjtu.edu.cn*

*or Hongmei Ma: mahongmei@pric.org.cn*

Table S4. Monte Carlo permutation test for the effect of factors on rare community structure (Number of permutations: 120).

| Factor | RDA1 | RDA2 | r^2^ | p-value |
| --- | --- | --- | --- | --- |
| TOC | -0.74958 | -0.66191 | 0.6558 | 0.32500 |
| Thickness | 0.81025 | -0.58609 | 0.9816 | 0.03333 |
| pH | -0.99999 | 0.00337 | 0.6778 | 0.35000 |
| Moisture | 0.00196 | -1.00000 | 0.2942 | 0.68333 |
| Distance | -0.80780 | 0.58946 | 0.9075 | 0.10000 |
