# Supplementary material for: Independent Shifts of Abundant and Rare Bacterial Populations across East Antarctica Glacial Foreland
Source: Front Microbiol. 2017 Aug 10;8:1534. doi: 10.3389/fmicb.2017.01534 (PMC5554324; doi:10.3389/fmicb.2017.01534)
Supplement: Supplementary file 5 [file Image_1.PDF]

Supplementary Information

Independent shift of abundant and rare bacterial populations across the glacial foreland in East Antarctica

Wenkai Yan[1], Hongmei Ma[2*], Guitao Shi[2], Yuansheng Li[2], Bo Sun[2], Xiang Xiao[1], Yu Zhang[3*]

[1] School of Life Sciences and Biotechnology, Shanghai Jiao Tong University, Shanghai, China

[2] SOA Key Laboratory for Polar Science, Polar Research Institute of China, Shanghai, China

[3] State Key Laboratory of Ocean Engineering, Shanghai Jiao Tong University, Shanghai, China

*** Correspondence:**

Yu Zhang: zhang.yusjtu@sjtu.edu.cn

or Hongmei Ma: mahongmei@pric.org.cn

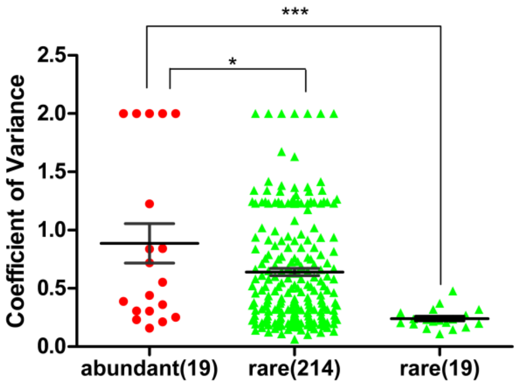

Figure S1. Coefficient of variation of abundant families and rare families at the five study sites with another tested cutoff (abundant: >1%, and rare: <1%). All 19 families of abundant bacteria, all 214 families of rare bacteria and the top 19 families of rare bacteria (ranked by the average proportion in the five sites) at the five study sites were analyzed. Unpaired t-test: ***, $p < 0.0001$; *, $p<0.05$). Bar: mean ± SEM.
