# Supplementary material for: Independent Shifts of Abundant and Rare Bacterial Populations across East Antarctica Glacial Foreland
Source: Front Microbiol. 2017 Aug 10;8:1534. doi: 10.3389/fmicb.2017.01534 (PMC5554324; doi:10.3389/fmicb.2017.01534)
Supplement: Supplementary file 7 [file Image_3.PDF]

Supplementary Information

Independent shift of abundant and rare bacterial populations across the glacial foreland in East Antarctica

Wenkai Yan[1], Hongmei Ma[2*], Guitao Shi[2], Yuansheng Li[2], Bo Sun[2], Xiang Xiao[1], Yu Zhang[3*]

[1] School of Life Sciences and Biotechnology, Shanghai Jiao Tong University, Shanghai, China

[2] SOA Key Laboratory for Polar Science, Polar Research Institute of China, Shanghai, China

[3] State Key Laboratory of Ocean Engineering, Shanghai Jiao Tong University, Shanghai, China

*** Correspondence:**

Yu Zhang: zhang.yusjtu@sjtu.edu.cn

or Hongmei Ma: mahongmei@pric.org.cn

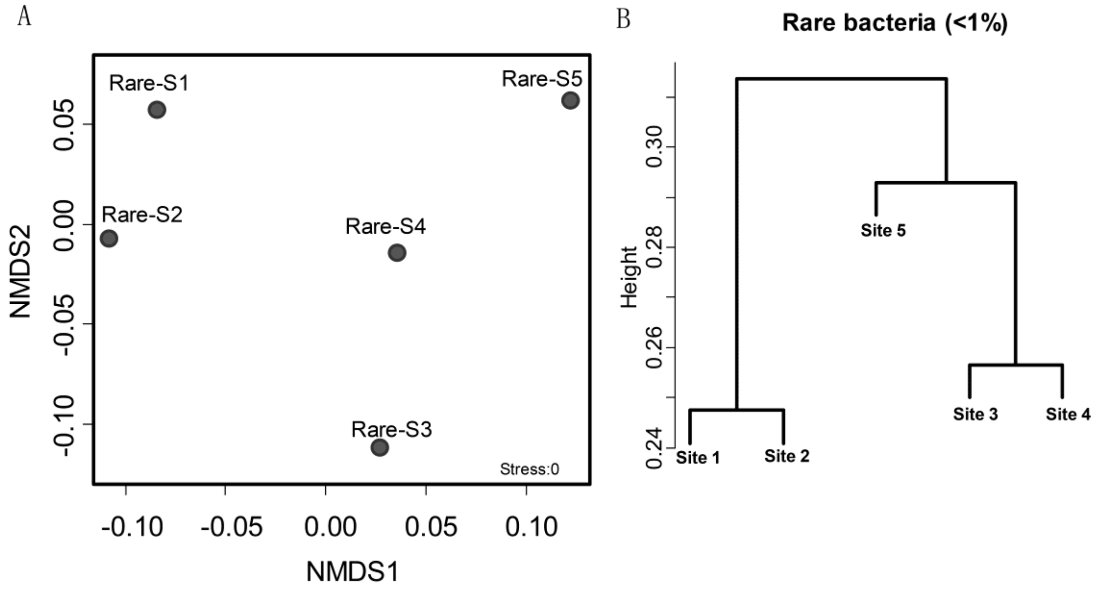

Figure S3. Clusters of rare families at the five study sites with another tested cutoff (rare <1%). (A)Non-metric multidimensional scaling (NMDS) of Bray-Curtis similarities of rare group at the family level in the glacial foreland. (B) A dendrogram analysis of the communities of rare group at the family level in the five glacial foreland soils.
