# Supplementary material for: Independent Shifts of Abundant and Rare Bacterial Populations across East Antarctica Glacial Foreland
Source: Front Microbiol. 2017 Aug 10;8:1534. doi: 10.3389/fmicb.2017.01534 (PMC5554324; doi:10.3389/fmicb.2017.01534)
Supplement: Supplementary file 9 [file Image_5.PDF]

Supplementary Information

Independent shift of abundant and rare bacterial populations across the glacial foreland in East Antarctica

Wenkai Yan[1], Hongmei Ma[2*], Guitao Shi[2], Yuansheng Li[2], Bo Sun[2], Xiang Xiao[1], Yu Zhang[3*]

[1] School of Life Sciences and Biotechnology, Shanghai Jiao Tong University, Shanghai, China

[2] SOA Key Laboratory for Polar Science, Polar Research Institute of China, Shanghai, China

[3] State Key Laboratory of Ocean Engineering, Shanghai Jiao Tong University, Shanghai, China

*** Correspondence:**

Yu Zhang: zhang.yusjtu@sjtu.edu.cn

or Hongmei Ma: mahongmei@pric.org.cn

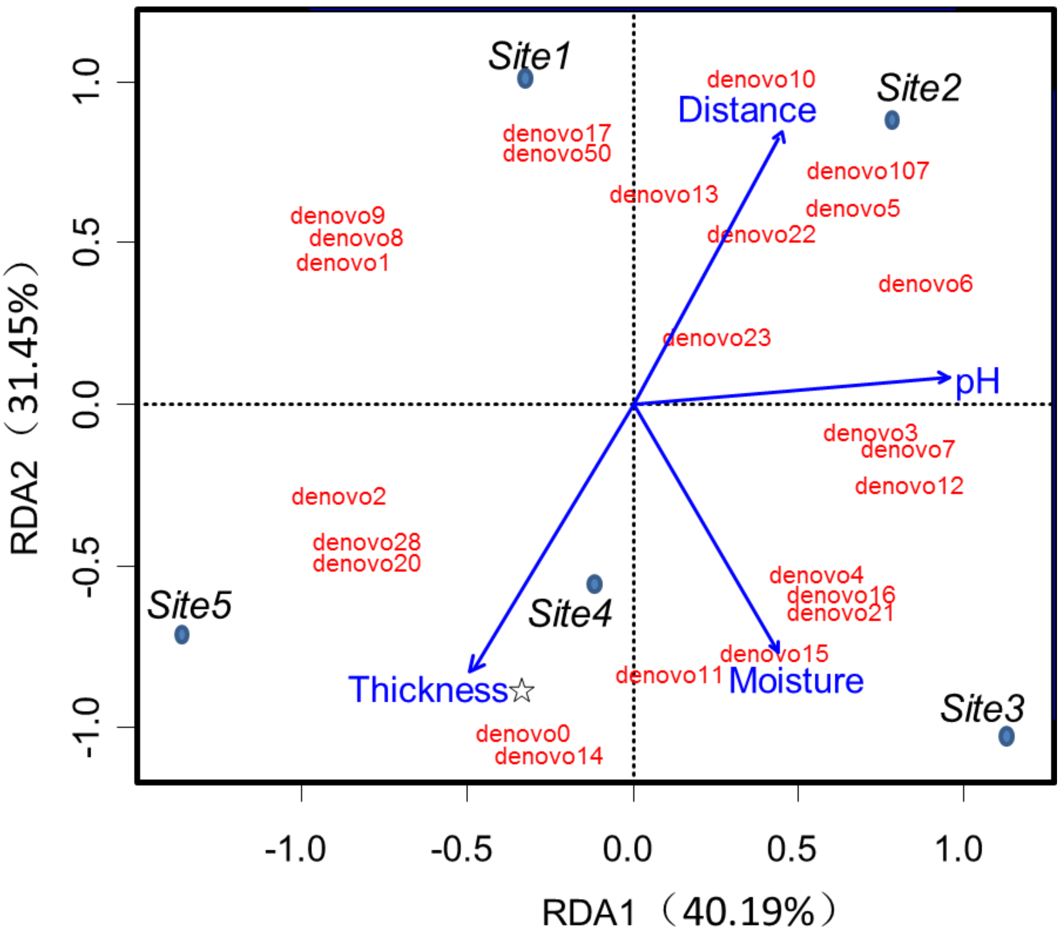

Figure S5. Ordination diagram of the RDA of abundant bacteria OTU profile in the glacial foreland. Blue dots indicate the study sites. Sites 1 and 2 were ice-free, and Sites 3, 4, and 5 were ice-covered. Red text indicates the abundant bacteria OTUs. Blue arrows indicate the environmental variables. The star indicates that the p-value <0.05 (Monte Carlo permutation test, Table S3). Distance was included here and did not affect the abundant bacterial group significantly (Monte Carlo permutation test, r^2=0.8884, p-value>0.05. Table S3).
