# Supplementary material for: Independent Shifts of Abundant and Rare Bacterial Populations across East Antarctica Glacial Foreland
Source: Front Microbiol. 2017 Aug 10;8:1534. doi: 10.3389/fmicb.2017.01534 (PMC5554324; doi:10.3389/fmicb.2017.01534)
Supplement: Supplementary file 10 [file Image_6.PDF]

Supplementary Information

Independent shift of abundant and rare bacterial populations across the glacial foreland in East Antarctica

Wenkai Yan[1], Hongmei Ma[2*], Guitao Shi[2], Yuansheng Li[2], Bo Sun[2], Xiang Xiao[1], Yu Zhang[3*]

[1] School of Life Sciences and Biotechnology, Shanghai Jiao Tong University, Shanghai, China

[2] SOA Key Laboratory for Polar Science, Polar Research Institute of China, Shanghai, China

[3] State Key Laboratory of Ocean Engineering, Shanghai Jiao Tong University, Shanghai, China

*** Correspondence:**

Yu Zhang: zhang.yusjtu@sjtu.edu.cn
or Hongmei Ma: mahongmei@pric.org.cn

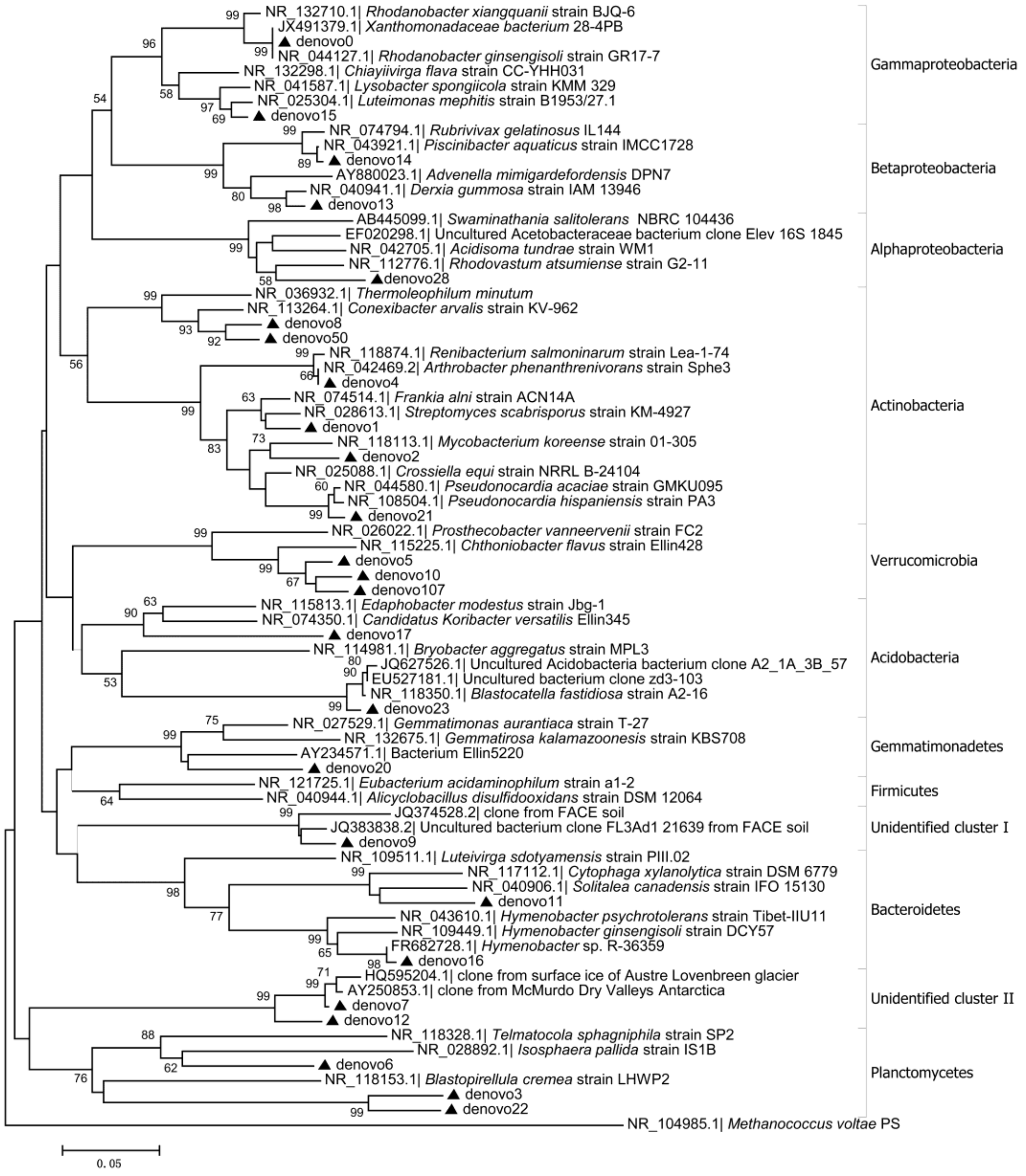

Figure S6. Phylogenetic tree of abundant bacteria OTUs in the glacial foreland. The OTUs were determined with 97 % similarity based on the 16S rRNA gene sequences. The sequences from this study (marked with triangles) and the reference sequences were aligned with ClustalW in MEGA6. The tree was constructed with a Neighbor-joining method with MEGA6.0. The scale bar represents a difference of 0.05 substitutions per site.
